# The estimation of reproductive values from pedigrees

**DOI:** 10.1093/beheco/arad049

**Published:** 2023-06-23

**Authors:** Mirjam J Borger, Jan Komdeur, David S Richardson, Franz J Weissing

**Affiliations:** Groningen Institute for Evolutionary Life Sciences, University of Groningen, P.O. Box 11103, 9700 CC, Groningen, The Netherlands; Groningen Institute for Evolutionary Life Sciences, University of Groningen, P.O. Box 11103, 9700 CC, Groningen, The Netherlands; Centre for Ecology, Evolution and Conservation, School of Biological Sciences, University of East Anglia, Norwich Research Park, NR4 7TJ Norwich, UK; Groningen Institute for Evolutionary Life Sciences, University of Groningen, P.O. Box 11103, 9700 CC, Groningen, The Netherlands

**Keywords:** fitness in a life history context, individual-based simulations, individual reproductive value, life-history model

## Abstract

Quantifying fitness is important to understand adaptive evolution. Reproductive values are useful for making fitness comparisons involving different categories of individuals, like males and females. By definition, the reproductive value of a category is the expected *per capita* contribution of the members of that category to the gene pool of future generations. Life history theory reveals how reproductive values can be determined via the estimation of life-history parameters, but this requires an adequate life-history model and intricate algebraic calculations. Recently, an alternative pedigree-based method has become popular, which estimates the expected genetic contribution of individuals to future generations by tracking their descendants down the pedigree. This method is versatile and intuitively appealing, but it is unknown if the method produces estimates of reproductive values that are accurate and precise. To investigate this, we implement various life-history scenarios (for which the “true” reproductive values can be calculated) in individual-based simulations, use the simulation data to estimate reproductive values with the pedigree method, and compare the results with the true target values. We show that the pedigree-based estimation of reproductive values is either biased (in the short term) or imprecise (in the long term). This holds even for simple life histories and under idealized conditions. We conclude that the pedigree method is not a good substitute for the traditional method to quantify reproductive values.

## Teaser

To study selection in natural systems, it is important to estimate fitness. “Reproductive value” is a good fitness measure, but it is difficult to quantify. Pedigree data may provide an outcome, as reproductive values can be estimated by tracking the descendants of an individual down the pedigree. By means of simulations, we show that this method is less suitable than often thought, as the estimates of reproductive value are either not accurate or not precise.

## INTRODUCTION

Quantifying fitness is important to understand how natural selection affects evolution. Estimating fitness in the wild is, however, a difficult task. Studies often measure survival, recruitment rate, or the number of offspring reaching independence as a proxy for fitness. In some well-monitored populations, lifetime reproductive success can be determined. Expected lifetime reproductive success is often a good proxy for fitness (Brommer et al. 2004), but even this measure is incomplete as it does not account for the rate of reproduction and the fact that different types of offspring (e.g., females and males; different size classes) cannot just be added up, as they have a different potential for spreading their genes to future generations.

Reproductive value (RV) (Fisher 1930) is a comprehensive fitness measure that copes with these problems. The reproductive value of a certain class of individuals is defined as the expected contribution of an individual in that class to the future gene pool of the population (Grafen 2006). Reproductive values are influenced by all kinds of life-history decisions, like age at first reproduction, trade-offs between survival and fecundity, risk-taking behavior, or the kind of offspring produced (e.g., sons or daughters). RVs are a particularly useful tool in behavioral ecology, as they allow for the quantification of the evolutionary costs and benefits of fitness-relevant individual decisions. For example, Tinbergen and Daan (1990) used an RV approach to predict the optimal clutch size in passerine birds on the basis of the trade-off between current and future reproduction. Reproductive values also play an important role in predicting the optimal sex ratio (Pen and Weissing 2002) and in the evolutionary theories of senescence (Hamilton 1966; Baudisch 2005).

While the definition of reproductive value is straightforward, its measurement is not. As briefly reviewed below, RVs can be derived from a life-history model by solving an eigenvector equation that contains all relevant life-history parameters (Caswell 1982). In line with this, most applications of RVs in empirical systems first make a life-history model, then estimate the parameters of this model, and finally obtain RVs by inserting these parameters in the eigenvector equation (Leverich and Levin 1979; Schulman and Chapais 1980; Tinbergen and Daan 1990; Newton and Rothery 1997; Pen et al. 1999; Bonduriansky and Brassil 2002; van de Pol et al. 2007; Bouwhuis et al. 2012). Hereafter, we will call the RVs thus obtained “model-based reproductive values” (mRV). Obtaining a good estimate of reproductive values along these lines can, however, be difficult in natural systems, since it requires a good understanding of all relevant life-history transitions and because life-history parameters can often only be estimated with limited accuracy and precision.

Recently, reproductive values have been estimated with a more intuitive method, based on genetic pedigree data (Barton and Etheridge 2011; Chen et al. 2019; Hunter and Slate 2019; Hunter et al. 2019; Reid et al. 2019; Alif et al. 2022). In this method, the average *per capita* number of descendants of the members of a certain class of individuals (e.g., immigrants into the population) down the pedigree is used as an estimate of the reproductive value of that class. Hereafter, we will call the RVs thus obtained “pedigree-based reproductive values” (pRV). The pRV method closely reflects Fisher’s definition of reproductive value. This method requires the availability of sufficiently deep and complete pedigrees, but if such pedigrees are available, it has the big advantage that no detailed knowledge of the underlying life-history model or estimates of life-history parameters are required. Therefore, the pRV method could potentially be a useful substitute for the mRV method. However, it still has to be determined whether the pRV method yields accurate and precise estimates of fitness differences when applied to field data.

In this theoretical study, we will investigate the performance of the pedigree method when applied to organisms with a relatively simple life history. To this end, we use individual-based simulations to produce several generations of individuals on the basis of a life-history model. The simulation data can be used to construct a pedigree (complete or incomplete) and to estimate life-history data such as survival probabilities and fecundities. Subsequently, reproductive values can be estimated by both methods (mRV and pRV) and compared with the “true” reproductive values (which are well-defined for the simulated populations). This allows us to judge the accuracy and precision of both estimates and to address questions like: how deep and complete must a pedigree be in order to get a reliable pRV estimate? How well must the life-history model be known, and how well do we need to know the life-history parameters in order to get reliable mRV estimates?

Before approaching the estimation problem, we first give some theoretical background by briefly reviewing some basal insights of life history theory.

## THEORETICAL CONSIDERATIONS: WHY ARE REPRODUCTIVE VALUES USEFUL?

In a population with discrete, non-overlapping generations and only one type of individual (e.g., no sex differences), expected lifetime reproductive success (ELRS) is an adequate fitness measure, as alleles that enhance the lifetime reproductive output of their bearers have a selective advantage. The situation is different in populations with different classes of individuals (e.g., females and males; breeders and helpers; workers, soldiers, and reproductives). In such cases, alleles inducing their bearers to produce a maximal number of offspring throughout their lifetime are not necessarily favored by selection. The reason is that different types of offspring may differ in the efficiency with which they spread genes to future generations. For example, in a population with a 4:1 female-biased sex ratio (four females per male), the reproductive success per male is on average four times greater than the reproductive success per female (because each offspring has one mother and one father). Therefore, an allele inducing the lifetime production of two sons has a lower ELRS than an allele inducing the lifetime production of four daughters, but a higher “fitness”: it spreads more efficiently to future generations, as the expected reproductive success of two sons is twice the expected reproductive success of four daughters (for the given sex ratio). Similarly, adding up potential offspring does not result in a suitable fitness measure when generations are overlapping and different age classes coexist. In this situation, the timing of reproduction matters: for example, it may be advantageous to produce offspring as early in life as possible (even if this leads to a lower lifetime production of offspring), as early-born offspring can more quickly contribute to the spread of their parental genes.

**Figure 1:**
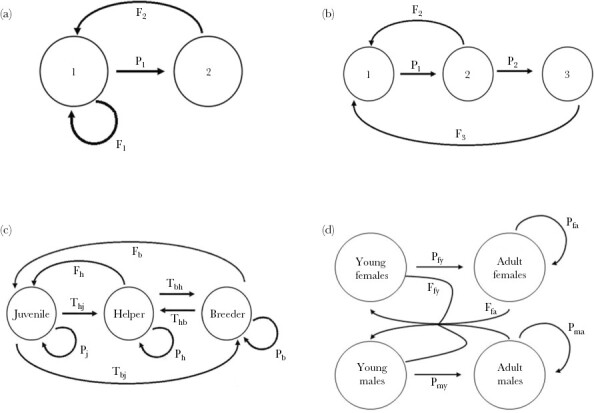
The four life-history scenarios considered in this study. (a) A two-stage life cycle with two age classes. One-year-old individuals survive to their second year with probability *P*_1_, second-year-old individuals die after reproduction. *F*_1_ and *F*_2_ denote the expected number of offspring (surviving to their first year) produced by 1- and 2-year-old individuals, respectively. (b) A three-stage life cycle with three age classes. Only 2- and 3-year-old individuals can reproduce (with expected fecundity *F*_2_ and *F*_3_), and 1- and 2-year-old individuals survive to the next year with probabilities *P*_1_ and *P*_2_, respectively. (c) A three-stage life cycle with behavioral stages. A juvenile can either stay juvenile for another time unit (probability *P*_j_), promote to helper status (probability *T*_hj_), promote to breeder status (probability *T*_bj_), or die. A helper can either stay a helper for another time unit (probability *P*_h_), promote to breeder status (probability *T*_bh_), or die. A breeder can stay a breeder (probability *P*_b_) or die. Both helpers and breeders can reproduce with expected fecundity (number of offspring surviving to the juvenile stage) *F*_h_ and *F*_b_, respectively. (d) A four-stage life cycle including differences between sexes. Young females and young males survive to become an adult with probabilities *P*_fy_ and *P*_my_, respectively. Adult females and adult males survive to the next year (and stay in the same state) with probabilities *P*_fa_ and *P*_ma_, respectively. Young females and adult females have expected fecundities of *F*_fy_ and *F*_fa_. They randomly pick a mate from the population of males. A newly produced offspring becomes a male with probability *s* and a female with probability 1-*s*, where *s* is the primary sex ratio.


Quantifying fitness in a life-history context (i.e., in a population with different categories of individuals) is therefore not an easy task. Fortunately, life-history theory is a well-developed branch of evolutionary biology (Roff 1992; Stearns 1992; Caswell 2001). For simplicity, we here focus on the special case of a discrete time structure (i.e., time is proceeding in discrete steps from one life-history event to the next) and a finite number of categories of individuals (e.g., males and females). In this case, a typical life-history model starts with a life-cycle graph (see Figure 1 for examples) that includes all life history “stages” (e.g., all age classes) and the transitions between these stages (e.g., survival to the next age class; production of offspring of age one). This graph can be translated into a “stage transition matrix” which in the case of three stages looks like this:

$$\begin{array}{l} A=\left(\begin{array}{lll} a_{11} & \;a_{12} & \;\;a_{13}\\ a_{21} & \;a_{22} & \;a_{23}\\ a_{31} & \;a_{32} & \;a_{33} \end{array}\right) \end{array}$$ (1)

Matrix element $a_{ij}$ is the *per capita* contribution of a member of stage *j* to the number of individuals in stage *i* in the next time step. For example, the stage transition matrix of an age-structured population with three age classes has the special form of a “Leslie matrix”:


$$\begin{array}{l} A=\left(\begin{array}{lll} F_{1} & \;\;F_{2} & \;F_{3}\\ P_{1} & \;0 & \;0\\ \;0 & \;P_{2} & \;0 \end{array}\right). \end{array}$$ (2)

Here, $P_{1}$ is the probability to survive from age class 1 to age class 2, $P_{2}$ is the probability to survive from age 2 to age 3, and $F_{1},\;F_{2},\;\;and\;F_{3}$ are the expected numbers of surviving offspring (surviving till age class 1) produced per individual of the three age classes.

Life-history matrices are a very useful tool, as they determine the dynamics of a stage-structured population (Stearns 1992). In most cases, such a population will converge to a “stable stage distribution” (in the case of an age-structured population: a stable age distribution; in the case of a population with two sexes: a stable sex ratio), that is, to a state where the ratio between the number of individuals in the stages ($n_{1}:n_{2}:n_{3}$) does not change anymore. Once the stable stage distribution is reached, the population as a whole grows with a characteristic “population growth factor λ.” When λ = 1, the population size will stay constant over time, while the population size will increase exponentially when λ > 1 and decrease exponentially when λ < 1.

The population growth factor λ is the most fundamental fitness measure in a stage-structured population (Brommer 2000): if we consider a population with transition matrix **A** and corresponding λ, a mutant inducing a slightly different transition matrix $A_{m}$ with growth factor $\lambda_{m}$ can be expected to successfully invade the population if $\lambda_{m}>\lambda$, while mutants with $\lambda_{m}<\lambda$ will go extinct. Mathematically, λ is the “dominant eigenvalue” of the stage-transition matrix **A**, and there are recipes to calculate λ for a given matrix **A**. Unfortunately, the eigenvalue equation determining λ is an “implicit” equation (in case of an age-structured population the so-called “Euler-Lotka equation”; Otto and Day 2007), which even for simple life histories can only be solved numerically and does not provide much insight into the factors governing evolution. It is therefore useful to look out for an alternative way to quantify fitness.

At this point reproductive values come into play. Conceptually, the “reproductive value” of a certain type of individuals (e.g., immigrants) is the expected genetic contribution of an individual of this type (e.g., of an immigrant) to the gene pool of future generations (Grafen 2006). Here, we will mainly apply the concept to situations where the “type” of an individual corresponds to a stage of a life-history model: the reproductive value *v*_*i*_ of stage *i* is the expected genetic contribution of a member of stage *i* to the gene pool of future generations. We will argue later that our results are of broader relevance than this. Mathematically, the vector $v^{T}=(v_{1},v_{2},v_{3})$ of reproductive values satisfies the equation $v^{T}\cdot A=\lambda \cdot v^{T}$, which means that it is a “left eigenvector” with respect to the dominant eigenvalue λ. Reproductive values are useful for making evolutionary predictions in two different ways. First, in many life-history contexts, including those of age-structured populations, natural selection tends to maximize the reproductive value of each age class (Schaffer 1974). This is the basis of dynamic programming (Houston and McNamara 1999), a powerful technique for solving difficult problems in behavioral ecology, such as finding the optimal allocation of resources to various activities, like growth and reproduction. Second, and more important for us, the reproductive values in a given “resident” population can be used to calculate the “selection gradient” ∂λ/∂x, which tells us whether the population growth factor λ (the most fundamental measure of fitness) will increase or decrease with a change in a (behavioral) strategy *x*. In other words, the selection gradient tells us the direction of selection: if ∂λ/∂x is evaluated at the strategy x* of the resident population, a positive selection gradient indicates that larger values $x>x^{*}$ are selectively favored, while smaller values $x<x^{*}$ are favored in the case of a negative gradient. Although λ is very difficult to calculate as a function of *x*, the sign of the selection gradient can relatively easily be determined by the following equation (Taylor 1990; Taylor and Frank 1996; Brommer 2000; Pen and Weissing 2000b, 2002; Otto and Day 2007; Lion 2018):


$$\begin{array}{l} sign\;\left(\frac{\partial \;\lambda }{\partial \;x}\right)=sign\;\left(\sum_{i,j}\;v_{i}\cdot \frac{\partial \;a_{ij}}{\partial \;x}\cdot n_{j}\right). \end{array}$$ (3)

Here, the *v*_*i*_ and the *n*_*i*_ are the reproductive values and the relative abundances of the various stages in the resident population (at demographic equilibrium) and the derivatives are evaluated at $x=x^{*}$.

Why is an equation like this useful? Assume that we want to know whether, in an age-structured population, selection will favor an increase in the reproductive effort at age *i*. To this end, let *x* denote a behavioral strategy that enhances the reproductive output at age *i* with a rate $\partial \;F_{i}/\partial x=b$ but reduces survival to the next age with a rate $\partial \;P_{i}/\partial x=-c$, where *b* and *c* indicate the “benefit” and the “cost” of an increase in *x*. Now the sum on the right-hand side of (3) has only two terms, and the sign of the selection gradient is readily obtained


$$\begin{array}{l} sign\;\left(\frac{\partial \;\lambda }{\partial \;x}\right)=sign\;\left(v_{1}\frac{\partial \;F_{i}}{\partial \;x}n_{i}+v_{i+1}\frac{\partial \;P_{i}}{\partial \;x}\cdot n_{i}\right)=sign\;\left(b\;v_{1}-c\;v_{i+1}\right). \end{array}$$ (4)

Hence, the selection gradient is positive if $b\;v_{1}>c\;v_{i+1}$, or equivalently:


$$\begin{array}{l} b/c>v_{i+1}/\;v_{1}. \end{array}$$ (5)

This inequality is known as the “asset protection principle”: the age classes with the highest future fitness expectation $v_{i+1}$ (i.e., those age classes that have “much to lose”) should be least inclined to take the survival risks associated with increasing the reproductive effort (see Wolf et al. 2007 for various implications of this principle). We will apply this principle in one of our examples below.

As illustrated by these calculations, reproductive values are useful tools in translating intricate fitness considerations into transparent cost-benefit comparisons (see Pen and Weissing 2000a, 2000b, 2002 for other examples). Cost-benefit considerations are further simplified by the fact that reproductive values are *relative* attributes that are only determined up to a constant factor. This implies that they can be normalized in the most convenient manner, for example by equating one of the reproductive values to one (e.g., $v_{1}=1$, which would simplify Equation 5) or by equating the sum of all reproductive values to one.

Finally, we would like to remark that classical life history theory is ecologically not consistent, in that its models predict exponential population growth (when λ > 1) or exponentially population decline (when λ < 1). To cope with this problem, density dependence needs to be incorporated into a life history model. This has to be done in an explicit manner, as the form of density dependence can have major implications for the course and outcome of evolution (Mylius and Diekman 1995; Pen and Weissing 2000b). Below we illustrate how to introduce density dependence in a life-history model. Rather than complicating the model, this actually simplifies calculations, as the inclusion of density dependence has two benefits: (1) it allows the assumption that λ = 1 at ecological equilibrium and (2) it reduces the dimensionality of the parameter space (thus making it easier to classify the evolutionary outcomes).

## METHODS

### Individual-based simulations and the estimation of pRVs

All individual-based simulations were based on a specific life-history scenario. Each scenario is specified by a set of parameters (such as the age-dependent survival probabilities *P*_*i*_ and fecundities *F*_*i*_ characterizing an age-structured population). For a given state *X*, all individuals in that state have the same state-specific parameters. Accordingly, all individuals in that state have the same (expected) state-specific reproductive value. Time proceeds in discrete steps, where one time step corresponds to a reproductive season. During a time step *t*, each individual is in a certain state (either an age class or a breeding state), which can change from one time step to the next. Reproduction takes place at the start of each time step. If, according to the life history scenario, *F*_*X*_ is the expected fecundity in state X, the individuals in that stage produce *F*_*X*_ offspring on average, while the actual number of offspring produced per individual is drawn from a Poisson distribution with mean *F*_*X*_, reflecting a standard assumption in population genetics (Crow and Kimura 1970). For simplicity, we assumed asexual reproduction and haploid individuals in most of our simulations; yet see scenario 4 for sexual reproduction with diploid individuals. Offspring enter the population at time *t*+1, and they belong to age class 1 in the age-based scenarios and to the juvenile state in the breeder-helper scenario. After reproduction, all individuals present at time *t* change their state stochastically (including death and staying in the same state), according to the probability assigned to their state by the life-history model. Each simulation was repeated for a fixed number of time steps *T* (typically *T* = 100, of which the first 20 time steps are shown in the figures). For each life-history scenario, simulations were run for various parameter combinations, and in each case the simulations were repeated at least 100 times. To ensure that our results were not biased by start-up effects, we initialized all populations in demographic equilibrium. Our results are therefore not examples of “transient dynamics” (Hastings 2004; Hastings et al. 2018). This was confirmed by additional simulations that were first run for thousands of time steps (thus ensuring equilibration) before the measurements were started.

We used a “gene-dropping” approach (MacCluer et al. 1986) to estimate pRVs. At the start of the simulation (*t* = 0), every individual is endowed with a unique marker (corresponding to a unique allele at a locus with infinite alleles). To ensure that gene dropping happened in demographic equilibrium, we first let each simulation run for 100 time steps (from *t* = −100 until *t* = −1) before gene dropping was initiated (at *t* = 0). At each reproduction event, the offspring inherit their marker from their parent. This allowed us to identify the descendants of each individual of the initial population in all future time steps. For each life history state *X* and each time *t*, we determined the average *per capita* number of descendants at time *t* for those individuals in the initial population that started in state *X*. We interpret this number, $pRV_{X}(t)$, as the pedigree-based estimate of the RV of individuals in state *X*: according to the definition of RV (“expected *per capita* contribution to the gene pool of future generations”) $pRV_{X}(t)$ should, for large *t*, approximate the “true” reproductive value of state *X*. We then normalize all reproductive values so that the RV of the “first” state (age class 1 in the age-structured scenarios; juvenile state in the helper-breeder scenario; young female in the sex-and-age-structured scenario) is scaled to 1. To achieve this, the values $pRV_{X}(t)$ were divided by $pRV_{1}(t)$, the estimated reproductive value of the first state. As shown in Supplementary Appendix A, the expected value of $pRV_{X}(t)$ can be calculated mathematically.

To prevent exponential growth or exponential decline of the simulated populations, we made the reasonable assumption that population sizes are kept in check by density dependent processes. Therefore, we added density regulation by assuming that fecundity is density dependent:


$$\begin{array}{l} F_{X}(N)=\frac{F_{X,0}}{1+\alpha N}, \end{array}$$ (6)

Where $F_{X,0}$ is the baseline fecundity in state *X*, *N* is population size and α indicates the intensity of density dependence. In other simulations (not shown), we also implemented other forms of density regulation (via density-dependent fecundity of only a single state or via density-dependent survival of one or all states); in all cases, the results agreed with those shown below. Unless indicated otherwise, we chose the parameter α in such a way that the population size of the resulting stationary population was *N* = 1,000. Note that this means that about 1,000 individuals are present at every time step. If *T* is the depth of the pedigree and *E* is the life expectancy of individuals, this implies that the pedigree encompasses about (T/E)⋅1,000 individuals. Thus, in all our simulations with a time horizon T=20 at least 10,000 individuals were included in the simulation-based pedigree.

### Estimation of mRVs

For the model-based estimation of reproductive values (mRV), the life-history parameters were estimated from the life-history events (survival, death, state transition, offspring production) observed in the same simulations from which the pRV estimates were derived. To estimate $mRV_{X}(t)$ for a certain time *t*, we used all events observed on individuals in state *X* up to time *t* to estimate the life-history parameters relevant for that state by averaging. For example, the fecundity *F*_*x*_ in state *X* was estimated by the average number of offspring produced in state *X* (per time step) by all individuals that had ever been in state *X* until time *t*. Subsequently, the $mRV_{X}(t)$ were estimated by calculating the left eigenvector of the matrix **A**(t) that is characterized by all parameter estimates up to time *t*. Obviously, a longer time horizon *t* provides a better estimate of the reproductive value of state *X*. However, it turned out that in all scenarios considered already in the first time step (i.e., in a single year) all life history parameters could be estimated sufficiently well to make $mRV_{X}(1)$ an excellent estimate of the RV of state X.

### Four scenarios

In this manuscript, we focus on four life history scenarios, which will be described in detail below. The life-cycle graphs of these scenarios can be found in Figure 1 and the transition matrices and calculations on reproductive value can be found in Supplementary Appendix B.

#### Scenario 1—A population with two age classes

This simple scenario, with only two life-history states and three life-history parameters, is characterized by Figure 1a. Both 1- and 2-year-old individuals can reproduce (with fecundity *F*_1_ and *F*_2_). One-year-old individuals survive to their second year with probability P_1_; all individuals die after their second year. A simple calculation (see Supplementary Appendix B) reveals that a stationary population (λ = 1) is only achieved if the life-history parameters satisfy the requirement:


$$\begin{array}{l} F_{1}+P_{1}\cdot F_{2}=1. \end{array}$$ (7a)

This equation makes intuitive sense: the left-hand side corresponds to the expected lifetime production of surviving offspring, which needs to be 1 in a stationary population. Condition (7a) for “ecological consistency” will not be satisfied for arbitrary values of the life-history parameters. However, it will eventually be satisfied, as, according to Equation 6, the fecundities decline with population density.

As shown in Supplementary Appendix B, the “true” reproductive values are given by:


$$\begin{array}{l} v_{1}=1,\;\;v_{2}=F_{2}. \end{array}$$ (7b)

#### Scenario 2—A population with three age classes

This scenario, with three life-history states and four life- history parameters, is described in Figure 1b. Two- and three-year-old individuals can reproduce (with fecundity $F_{2}$ and $F_{3}$). One- and two-year-old individuals can survive to their next year (with probabilities $P_{1}$ and $P_{2}$); all individuals die after their third year. Now, the condition for a stationary population and the reproductive values in that population are given by:


$$\begin{array}{l} P_{1}\cdot \left(F_{2}+P_{2}\;F_{2}\right)=1 \end{array}$$ (8a)

$$\begin{array}{l} v_{1}=1,\;\;v_{2}=F_{2}+P_{2}\;F_{3},\;\;v_{3}=F_{3}. \end{array}$$ (8b)

Ecological consistency (a stationary population) is ensured by Equation 6.

#### Scenario 3—A population with three behavioral classes

This scenario, which is characterized by Figure 1c, includes three life-history states (juveniles, helpers, breeders) and a full set of nine life-history parameters. All newly produced individuals start as juveniles. A juvenile either stays in juvenile state for another time period (with probability $P_{j}$), or it becomes a helper (with probability $T_{hj}$) or a breeder (with probability $T_{bj}$), or it dies (with probability $1-P_{j}-T_{hj}-T_{bj}$). Likewise, a helper either stays in the helper state for another period (probability $P_{h}$), or it becomes a breeder (probability $T_{bh}$), or it dies. A breeder either stays in the breeding state for another time step (probability $P_{b}$), or it loses its position and becomes a helper (probability $T_{hb}$), or it dies. Helpers and breeders can both produce offspring (fecundities $F_{h}$ and $F_{b}$ per time step).

#### Scenario 4—A population with both sex and age classes

This scenario, which is characterized by the life-cycle graph in Figure 1d, contains four life-history states: young females, young males, adult females, and adult males. Newly produced individuals start as young females or young males. The sex of an offspring is assigned at random, where the probability *s* of becoming a male corresponds to the primary sex ratio. The parameter *s* is the same for all individuals and constant throughout time. Young females survive to become adults with probability $P_{fy}$, and young males survive to become adults with probability $P_{my}$. Adult females and males survive to the next time step with probabilities $P_{fa}$ and $P_{ma}$, respectively. Both young and adult individuals can reproduce. We assume that each young female and each adult female produces on average $F_{fy}$ and $F_{fa}$ offspring per time step, where these fecundities are density dependent according to Equation 6. The realized number of offspring per female is drawn from a Poisson distribution. We assume that mating is at random and that, irrespective of its age, each male has the same probability of siring any given offspring: whenever an offspring was produced, a father was drawn at random from the set of all (young and adult) males.

### Technical note

Simulations were ran in Visual Studio Enterprise 2019 (version 16.8). Figures were made using R 3.4.1 (R Core Team 2021), with the packages ggplot2 (Wickham 2016) and cowplot (Wilke 2020).

## Results

### Importance of stochasticity

**Figure 2:**
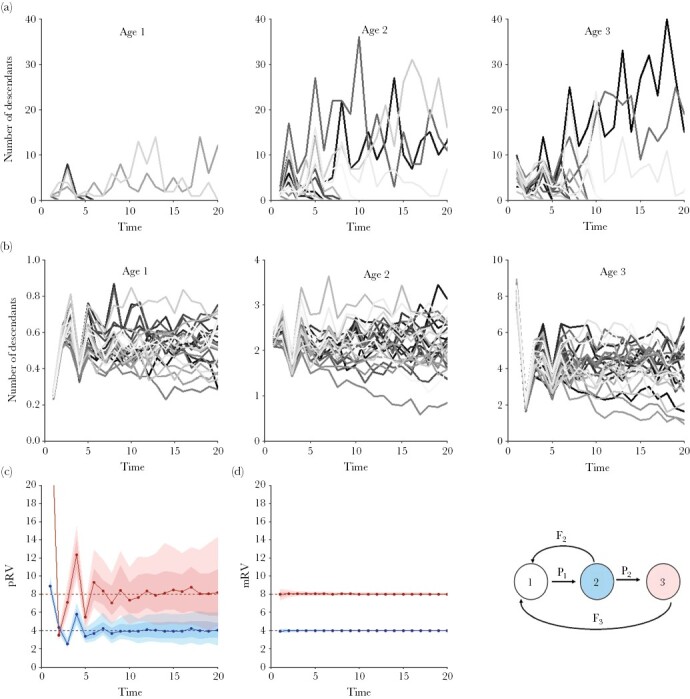
Stochasticity in the simulation outcomes. Example simulations based on Scenario 2 (three age classes) illustrate stochasticity within and across simulations. (a) Variation within a simulation. The number of descendants (down the pedigree) of 25 randomly chosen individuals per age class within the same simulation. There is considerable variation between individuals, but as it is purely caused by stochastic events (all individuals in an age class have the same life-history parameters), it is not meaningful from an evolutionary perspective. (b) Variation across simulations. The average number of descendants per age class in 25 randomly chosen simulations. Despite the fact that averages are based on hundreds of individuals per age class, there is still considerable variation across simulations. (c, d) Graphs summarizing the estimates of pRV and mRV for the same 100 replicate simulations. In both cases, RV_1_ was normalized to 1. For the parameter values of the simulation (*P*_1_ = 0.25, *P*_2_ = 0.25, *F*_2_ = 2, and *F*_3_ = 8), the “true” reproductive values of age classes 2 and 3 are *v*_2_ = 4 and *v*_3_ = 8 (see equation 8b); dashed horizontal lines in the graphs). The dots connected by solid lines indicate the median value of the simulations, the dark shades indicate the 50% central values and the lighter shades indicate the 90% central values of the simulations. (c) The pRV estimates are based on the average number of descendants per age class. (d) The mRV estimates are based on equation 8b, where the age-dependent survival probabilities and fecundities were estimated from the simulation data. Already after a single time step, the mRV estimates are quite accurate and precise.

For the case of a population with three age classes, Figure 2 illustrates various sources of stochasticity and the implications thereof for the estimation of reproductive values. Figure 2a shows that, within a single simulation, stochasticity in individual reproductive success is extensive. In each age class, the majority of individuals does not leave any descendants after ten time steps, while the few other individuals leave a large number of descendants. In a population of constant size, this is not surprising, but it is important to keep the huge variation in individual reproductive success in mind. For evolutionary considerations, estimating RV based on single individuals is therefore not very meaningful: these values mainly reflect stochasticity in realized long-term reproductive success, rather than the individuals’ capability of spreading their genes to future generations. It is, however, meaningful to estimate the RV of individuals having a certain property in common, such as age, sex, migration status, or personality. For the pRV method, this means that the reproductive success (down the pedigree) is averaged for a sufficiently large number of individuals sharing this property. For the property “age,” the result is shown in Figure 2b. The three panels illustrate that there is still substantial variation across replicate simulations, even though all individuals have exactly the same life-history parameters, all simulations start in demographic and ecological equilibrium, and mean reproductive success in each age class is based on averaging hundreds of individuals. As a field study on a single population is comparable to a single simulation, one should therefore expect considerable variation across populations, even if these populations are living under similar conditions.

### Estimation of pRVs in Scenario 2

Figure 2c illustrates the implications of stochasticity on the pedigree-based estimation of reproductive values. In the first ten time steps, the median pRV estimates of 100 simulations approach the “true” values (horizontal dashed lines) in a “zig-zag” manner. As shown in Supplementary Appendix A, this is not caused by short-term deviations from demographic equilibrium (“transient dynamics”; Hastings 2004; Hastings et al. 2018), but an intrinsic property of the inheritance dynamics in a class-structured population. Although the zig-zag pattern is to be expected in life-history models (see Supplementary Appendix A), it has the unfortunate implication that, in a short-time perspective, the pRV estimates deviate strongly and systematically from the true reproductive values. These “true” RVs (which in Scenario 2 are given by equation 8b) are asymptotic values, and we will see below that only these asymptotic values are relevant for evolutionary considerations. On a longer-term perspective (here: after about ten time steps), the median of the pRV estimates converges to the true RVs, but the individual estimates tend to differ considerably from the true values. In other words, the pRV estimates are systematically biased (and hence inaccurate) on a short-term perspective and imprecise on a longer-term perspective.

### Estimation of mRVs in Scenario 2

For the same simulations as in Figure 2c, Figure 2d illustrates the traditional model-based way of estimating RVs. To this end, the life-history parameters are estimated from the (simulation) data, and the mRVs are subsequently obtained by plugging in these estimates in equation 8b. Figure 2d reveals two things: First, the mRV estimates are highly accurate and precise. In other words, a single simulation (corresponding to a single field population) is sufficient to accurately derive reproductive values. Second, the mRV method is not “data hungry”: the data of a single time step are sufficient to obtain a rather accurate RV estimate. However, the mRV method crucially depends on the availability of a “correct” life-history model. We will later investigate a situation where the life-history model is incomplete.

### Estimation of pRVs in Scenario 1 (two age classes)

**Figure 3:**
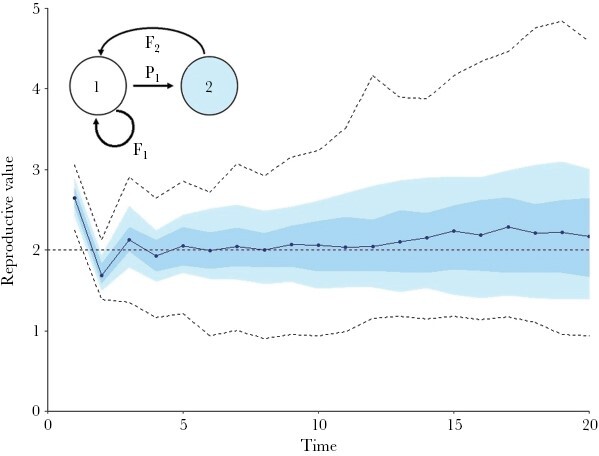
pRV estimates in Scenario 1 (two age classes). Summary graph of the (normalized) pRV estimates of the second age class in 100 replicate simulations. Graphical conventions are as in Figure 2c: the horizontal dashed line indicates the “true” RV of age class 2, and the dots connected by a solid line indicate the median of the pRV estimates. The dashed outer lines indicate the most extreme values of the 100 simulations. Parameter values: *F*_1_ = 0.5, *P*_1_ = 0.25, *F*_2_ = 2. In view of equation 7b, *v*_2_ = *F*_2_ = 2.

Figure 3 shows that the conclusions drawn above also hold for the simplest life-history scenario considered in our study. On a short-term perspective, a similar zig-zag pattern is observed, leading to a biased estimate of the “true” asymptotic RV *v*_2_. On a longer-term perspective, the pRV estimates of different simulations differ substantially from each other, implying that a single simulation (or, correspondingly, a single population in a field study) may give a wrong impression of the intensity and direction of selection.

### Estimation of pRVs in Scenarios 3 and 4

**Figure 4:**
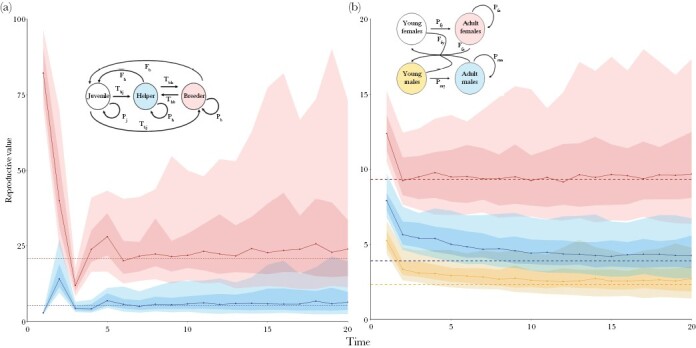
Pedigree-based estimates of RV in two more complex scenarios. Summary graph of the (normalized) pRV estimates in 100 replicate simulations based on (a) Scenario 3 (juvenile-helper-breeder), and (b) Scenario 4 (young and old males and females in a sexually reproducing population). The inserts show the life-cycle graphs. Graphical conventions are as in Figure 2c: the dashed lines indicate the true RVs, and the dots connected by a solid line indicate the median of the pRV estimates. In (a), blue represents the RV values of helpers and red the RV values of breeders. In (b), yellow represents the RV values of young males, while red and blue represent the RV values of adult females and males, respectively. Parameter values (a): *F*_*j*_ = 0, *F*_*h*_ = 0.05, *F*_*b*_ = 12.5, *T*_*hj*_ = 0.15, *P*_*h*_ = 0.2, *T*_*hb*_ = 0, *T*_*bj*_ = 0.01, *T*_*bh*_ = 0.2, *P*_*b*_ = 0.4 and (b): *P*_*fy*_ = 0.1, *P*_*fa*_ = 0.85, *P*_*my*_ = 0.5, *P*_*ma*_ = 0.9, *F*_*fy*_ = 0.095, *F*_*fa*_ = 2, *s* = 0.3.

Figure 4 illustrates that the problems with pRV estimation also arise in other life-history scenarios. In Scenario 3 (Figure 4a), the pRV estimates can be two or even three times as large as the true RVs in a considerable percentage of the simulations. Although the initial zig-zag pattern observed in Scenarios 1 and 2 does not appear in Scenario 4 (Figure 4b), pRV remains an biased estimator of the true (asymptotic) RV in the initial period; in fact, the systematic bias in this estimate only disappears after a long period (in this case 10 time steps). Hence, the simulations in Figure 4 confirm our earlier conclusions: in an initial time period, the pRV values (including their median) differ substantially and systematically from the true RVs. On a longer time horizon, the accuracy of pRV increases (the median pRVs approach the true RVs), but now the estimates are very imprecise, as the individual simulations differ considerable from each other. This results in the unfortunate conclusion that pedigree-based estimation of RVs is either inaccurate (in case of a shallow pedigrees including relatively few time steps) or imprecise (in case of deep pedigrees), even if the study population is relatively large (*N* = 1,000 in the simulations shown thus far), well-mixed, and in demographic and ecological equilibrium.

### Effects of population size and time scale

In Supplementary Appendix C, we also show the effects of population size and a longer time horizon on pRV estimates. When population sizes are small, pRV estimates are strongly affected by demographic stochasticity. After a while, all individuals present at a later time step are descendants of just one individual of the initial population. Accordingly, in each simulation, eventually pRV is equal to zero for all but one of the categories considered. Therefore, in small populations, the median pRV of 100 simulations converges to zero relatively rapidly (Supplementary Figure C1(a)). Furthermore, the variation of the pRV estimates across simulations increases with the time horizon (Supplementary Figures C1 and C2). In other words, the precision of pRV decreases with the depth of the pedigree.

### Evolutionary predictions based on RV

Above, we have seen that reproductive values are very useful for predicting the course and outcome of life history evolution. One might speculate that the large variance of pRV values that we observed in replicate simulations is associated with a corresponding variance in evolutionary outcomes. To check this, we added an evolving parameter *x* (corresponding to reproductive effort) to our Scenario 1 model with two age classes. To this end, we added a gene locus to the model, with a continuum of alleles *x*, ranging from *x* = 0 to *x* = 1. Depending on allele *x*, individuals have the parameters $P_{1}(x)=P_{1,0}\cdot (1-\frac{1}{2}\gamma x^{2})$, $F_{1}(x)=F_{1,0}\cdot (1+\beta x)$, and $F_{2}(x)=F_{2,0}=constant$, where β and γ are positive. Hence, 1-year olds with a larger reproductive effort *x* have a higher reproductive output *F*_1_, but a lower survival probability *P*_1_. We assume that *x* is transmitted from parent to offspring, subject to rare mutations with small effect size (mutation rate 0.01, mutational variance 0.0025; see Netz et al. 2022). In the course of the generations, *x* should evolve to a value that optimizes the balance between current and future reproduction. With the help of reproductive values, the optimal reproductive effort can easily be calculated. At evolutionary equilibrium, the selection gradient is zero, which according to Equation 4 corresponds to the equation $b/c=v_{2}/v_{1}\;\;$, where $b={F_{1}}^{\prime }(x)=F_{1,0}\beta$ and $c=-{P_{1}}^{\prime }(x)=P_{1,0}\;\gamma x$, while $v_{2}/v_{1}=F_{2,0}$, in view of equation 7b. Inserting all these terms and solving for *x*, we obtain the optimal reproductive effort


$$\begin{array}{l} x_{opt}=\frac{F_{1,0}}{P_{1,0}\cdot F_{2,0}}\cdot \frac{\beta }{\gamma }. \end{array}$$ (9)

Figure 6 demonstrates that, for a set of parameters yielding the optimal value $x_{opt}=0.5$, individual-based simulations do indeed converge to this value, and that they stay close to this value, irrespective of whether the pedigree-based estimate $pRV_{2}/pRV_{1}\;$ of $v_{2}/v_{1}\;$ is much larger or much smaller than the “true” value $F_{2,0}$. We conclude that (1) reproductive values are indeed useful for making evolutionary predictions; but that (2) pedigree-based estimates are too imprecise to be reliable predictors of the evolutionary outcome.

**Figure 5:**
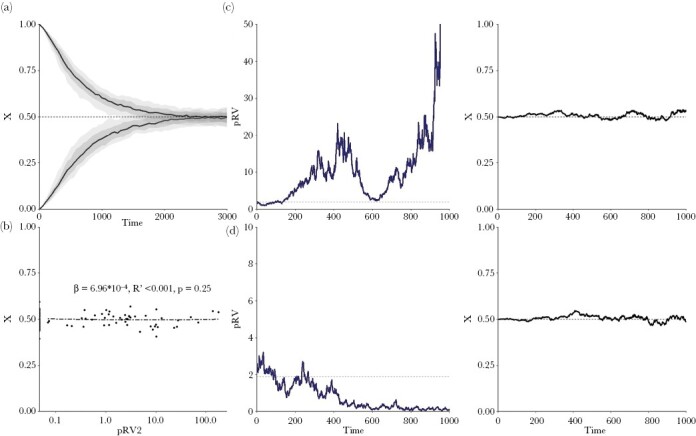
Evolutionary predictions based on pRVs. (a) Evolution of reproductive effort *x* in Scenario 1 (two age classes) in two sets of 100 individual-based simulations; one set starting at x=0 and the other starting at x=1. The solid lines indicate the median *x*-values of these simulations. The 50% (resp. 90%) central values of x are represented by the darker (resp. lighter) shaded areas around the medians. The dashed line indicates the reproductive effort $x_{opt}=0.5$, which, according to Equation 9, is evolutionarily optimal for the parameter setting $P_{1,0}=0.25,\;\;F_{1,0}=0.5,\;\;F_{2,0}=2,\;\;\beta =0.5,\;\;\gamma =1$. (b) Although the values of pRV_2_ estimates differ by orders of magnitude across simulations (horizontal axis), the average *x*-value evolved in these simulations at time step *t* = 1000 approaches $x_{opt}=0.5$ quite closely in all simulations (vertical axis). The regression line through the data is almost identical with the line x=0.5. In other words, the pRV estimates do not predict the outcome of evolution. **(c)** and **(d)** show two example simulations from (b), which both start at $x_{opt}=0.5$. The evolutionary trajectories of *x* stay close to this equilibrium value (dashed line), while the pRV ratio $pRV_{2}/pRV_{1}\;$ either overestimated (b1) or underestimated (c1) the “true” RV ratio $v_{2}/v_{1}\;$ (dashed line).

**Figure 6:**
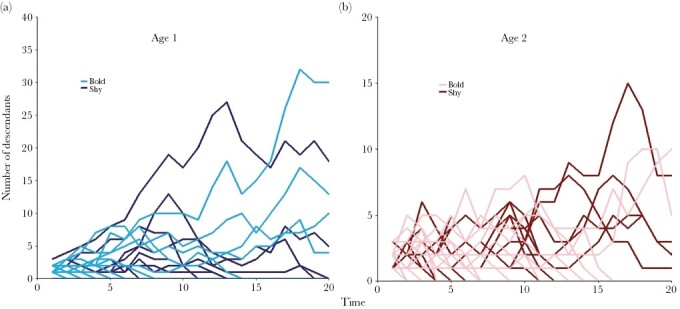
Number of descendants for individuals differing in a personality trait. The number of descendants of **(a)** 1-year-old and **(b)** 2-year-old individuals that are either bold (light color) or shy (dark color) in an illustrative simulation. Twenty-five randomly chosen individuals per age class and personality were followed. The underlying life-history model (which may be unknown to the researcher) has the parameters: *F*_*1s*_ = 0.2, *F*_*1b*_ = 0.5, *F*_*2s*_ = 0.8, *F*_*2b*_ = 1.8, *P*_*1s*_ = 0.7, *P*_*1b*_ = 0.5, *m* = 0.3. Hence, bold individuals have a lower survival, but a higher fecundity. The corresponding “true” RVs are: *v*_*1s*_ = 1, *v*_*1b*_ = 1.8, *v*_*2s*_ = 1.1 and *v*_*2b*_ = 2.3. Hence, bold individuals have a higher reproductive value than shy individuals. However, this difference is not apparent from the numbers of descendants in (a) and (b). The inspection of other simulations led to the same conclusion.

### Estimating RVs when the life-history model is wrong or incomplete

Until now, we had to conclude that the traditional model-based estimation of reproductive values is clearly superior to the pedigree-based method. However, the model-based method obviously relies on the existence of a good life-history model. In natural populations, it is often very difficult to decide which traits need to be taken into consideration, and how different life-history decisions relate to each other. In a situation like this, the “model-free” pedigree-based estimation of RVs may provide an outcome, as it has the potential to estimate reproductive values (and hence “fitness”) for all kinds of traits of interest. This way, a pedigree-based approach may be capable to pick up signals that certain traits are fitness-relevant and should be incorporated in a life-history model. Here, we illustrate this by reconsidering our model in Scenario 1 (two age classes) and assuming that, for a given population, this model is incomplete as it neglects “personality” differences, while such differences are fitness-relevant (e.g., Wolf et al. 2007; Réale et al. 2009; Cole and Quinn 2014; Ward-Fear et al. 2018). Is the pedigree method able to quantify the effect of personality on RVs?

To investigate this, we extended the life-history model of Scenario 1 in such a way that the individuals had either a “bold” or a “shy” personality (see Supplementary Appendix D for details). Researchers can notice these differences, but it is not known to them how boldness and shyness affect the life-history parameters. However, they can determine the number of descendants of bold and shy individuals down a pedigree, hoping to find out whether there is selection on personality. We, as the designers of the simulation program, know that bold individuals have a higher fecundity at both ages, that shy individuals have a higher survival probability from age 1 to age 2, and that the personality of an individual is randomly determined at birth (with *m* being the probability of being bold). We can also calculate that the RV of bold individuals is about twice as large as the RV of shy individuals. Figure 6 shows that, despite this large difference, the inherent stochasticity of the pedigree method does not easily allow to pick up the signal. We conclude that large sample sizes (i.e., large pedigrees) are needed to reliably determine fitness differences and selection differentials, and that differences in pRV values need to be interpreted with care.

## DISCUSSION

Reproductive values are a very useful theoretical tool, as they can help to answer evolutionary questions like: what is the optimal clutch size (Tinbergen and Daan 1990)? What is the optimal investment in male versus female offspring (Fisher 1930; Pen and Weissing 2002)? Under which circumstances should offspring stay on their natal territory and help their parents raise additional offspring (Pen and Weissing 2000c)? However, we need to be able estimate RVs with accuracy and precision if we are to use them to test evolutionary predictions.

Reproductive values are increasingly estimated by making use of pedigree information from long term studies (e.g., Barton and Etheridge 2011; Chen et al. 2019; Hunter and Slate 2019; Hunter et al. 2019; Reid et al. 2019; Alif et al. 2022). In essence, the *per capita* number of descendants of the members of a certain life-history stage is used to estimate the reproductive value of that stage. This method is intuitively appealing, as it closely reflects Fisher’s (1930) definition of “reproductive value.” However, our study clearly reveals that the method has important drawbacks when used to estimate fitness, even if the pedigree includes many individuals (hundreds or even thousands) per time step. The analytical arguments (Supplementary Appendix A) and simulations outlined in the results show that pedigree-based estimates are strongly and systematically biased when estimating asymptotic RVs (which are the relevant entities in evolutionary considerations) if the pedigree encompasses a short time horizon (say, t<10). On a longer time horizon, the median pRVs of 100 replicate simulations match the “true” asymptotic RVs reasonably well, but the individual simulations tend to diverge considerably from each other with increasing “depth” of the pedigree. As one simulation corresponds to one field study, this implies that the pedigree-based estimate of RV is typically way “off target” if it is only based on one, or a small number of, field studies.

Our findings concur with the patterns reported in natural populations. In both Soay sheep (*Ovis aries*, Hunter et al. 2019) and Florida Scrub-Jays (*Aphelocoma coerulescens*, Chen et al. 2019) estimates of RV based on individuals did not stabilize, just as in our simulations. Moreover, the pedigree-based estimates showed a similar initial “zig-zag” pattern as in our study (e.g., Figures 2c, 3, 4a). Last, but not least, when the estimation was repeated for the same population by applying the gene-dropping method to different cohorts, the RV estimates obtained varied a lot (Hunter et al. 2019).

Our study confirms that the traditional, model-based calculation of reproductive values on the basis of estimates of the life-history parameters has more desirable statistical properties. In all our simulations, we found that already after one or two time steps, the life-history parameters could be estimated sufficiently well to estimate asymptotic RVs with high accuracy and precision. The mRV method has the additional advantage that a sensitivity analysis (Caswell 2019) can reveal how parameter uncertainty affects the precision of the estimate. In the field, such uncertainty can be considerably larger than in our simulations. For example, we assumed that all stage transitions, including death, can be observed in the study population. In real populations, the estimation of, say, survival probabilities will often be unprecise and/or biased, as mortality cannot always be distinguished from emigration. However, the mRV method has the major drawback that it relies on a sound life-history model, including all fitness-relevant aspects, which in practice will often not be available. We have the impression that in the literature the pRV method is often used to fill this gap, as it allows to estimate reproductive values of states or traits that are not (yet) incorporated in an established life-history model. Although this may be a useful first step, the results of such a pedigree-based analysis should not be overinterpreted. As illustrated in Figure 6, the stochasticity inherent in the estimation of pRVs makes it very difficult to draw reliable conclusions on fitness differences and selection gradients.

We would like to emphasize that our disappointing conclusions on the pRV method mainly relate to evolutionary considerations, which are usually based on asymptotic reproductive values. In other contexts, a pedigree analysis based on gene dropping can be a useful tool, for example to understand whether frequencies of certain rare diseases are caused by founder effects (O’Brien et al. 1988), to better understand genetical processes in rare species to improve conservation management (Caballero and Toro 2000), or to study the effect of demography on the dynamics of short term genetic contributions (our “zig-zag” patterns).

We purposely kept our model assumptions as simple as possible. We focused on very simple life-histories, assuming that estimation issues that arise in simple scenarios will most likely be even worse in more complex life-histories. We assumed that our populations are closed (no emigration or immigration), that pedigree information is complete, and that the life-history parameters remained constant over time. Again, it is likely that RV estimates get worse when the situation is less ideal (as is the case for most field studies). Most of our simulations assume asexual reproduction. This has the advantage that pRV estimates can directly be based on the number of descendants, as in this case, it is equivalent to the expected genetic contributions (“the expected number of copies of an allele that an individual leaves in distant future generations, conditional of its pedigree of descendants,” Barton and Etheridge 2011). However, in sexual populations, these two quantities differ slightly. Yet, as shown in Figure 3b, our conclusions also apply to populations with sexual reproduction and different sexes. However, they are not necessarily representative for more complicated situations, such as kin interactions, inbreeding or inbreeding avoidance, and sexual selection. But again, we would argue that methods that do not work well in a simple context will most likely also fail in more intricate situations. Additionally, we would like to point out that the definition of individual reproductive value is not straightforward, as seemingly subtle differences exist in the literature (e.g., Engen et al. 2009 vs. Barton and Etheridge 2011), which seem to be used for different purposes and could lead to vastly different conclusions when mixed up.

Life-history theory is one of the most advanced branches of evolutionary biology, with sophisticated tools and methods and a well-established fitness concept (Brommer 2000). Individual-based simulations are rarely used in life-history studies, perhaps because analytical techniques are readily available. Yet, such simulations can provide valuable additional information. First, simulation models are very flexible and can easily be tailored to the intricacies encountered in real-world situations. For example, it is quite difficult to include sex- and age-structure, non-random mating, and kin interactions in a life history model without compromising analytical tractability, while this is straightforward in a simulation approach. Second, running replicated simulations gives researchers a good idea on the kind and degree of variation to be expected. Individual-based simulations are therefore a useful tool for judging the validity of empirical methods, such as the pedigree-based estimation of reproductive values.

## Supplementary Material

arad049_suppl_Supplementary_Material

## Data Availability

Analyses reported in this article can be reproduced using the data provided by Borger et al. (2023).

## Notes

We thank the MARM research group, the Seychelles warbler research group, and the reviewers of previous versions of this manuscript for their insightful comments on our study and the manuscript.
